# Ornamental *Phoenix* palm trees as habitat for fauna in the Mediterranean Region – results from a full year monitoring

**DOI:** 10.3897/BDJ.12.e123144

**Published:** 2024-05-17

**Authors:** Silke Laucht, Kaat Brulez, Jörg Hanisch, Alexander Blakey, Gabe Weyman, Jan-Dieter Ludwigs, Tania Alvarez

**Affiliations:** 1 RIFCON GmbH, Hirschberg, Germany RIFCON GmbH Hirschberg Germany; 2 Syngenta Limited, Bracknell, United Kingdom Syngenta Limited Bracknell United Kingdom; 3 Gabe Weyman Consulting Limited, Stockport, United Kingdom Gabe Weyman Consulting Limited Stockport United Kingdom

**Keywords:** *Phoenix*, palm trees, fauna, richness, vertebrates, invertebrates, ecological monitoring, seasonal cycle

## Abstract

In the European Mediterranean Region, palm trees are a common element in cities and semi-urban landscapes and have become important habitat structures for local fauna. This study aimed to monitor the invertebrate and vertebrate fauna occurring on and associated with ornamental palms of the genus *Phoenix*, over the course of one year. Five study sites were used in southern Spain, with varying levels of management. Several complementary methods were applied monthly in order to assess the vertebrates and invertebrates associated with the full seasonal cycle of palms, including flowering and fruiting. The study resulted in the identification of 216 invertebrate families from seven different classes and 89 vertebrate species, consisting of 62 bird, 20 mammal (including bats), six reptile and one amphibian species associated with *Phoenix* palms. It thus highlights that *Phoenix* palms provide a habitat for many species and individuals over the course of one year.

## Introduction

In the European Mediterranean Region, palm trees are a common element in cities and semi-urban landscapes and have become important habitat structures. Several species of palm trees are planted for ornamental reasons in gardens and parks, used as physical separation in agricultural environments or for fruit production (including now-abandoned palm groves).

There are 2,533 palm species recognised worldwide at the time of the study (Govaerts et al. 2023), only two of which are native to continental Europe: Cretan date palm *Phoenixtheophrasti* and European fan palm *Chamaeropshumilis* (*Johnson 1996*). However, the most common palm species in Mediterranean Europe are of the *Phoenix* genus, these being the date palm (*P.dactylifera*) and the Canary palm (*P.canariensis*), due to their uses in fruit production and ornamental use. In Spain, *P.dactylifera* covers around 500 hectares of agricultural land (Food and Agriculture Organization of the United Nations (FAO) 2023). Information on the number and distribution of palm trees present in non-agricultural scenes (e.g. as ornamental trees) is scarce. Using remote sensing on aerial images from the Alicante Province in Spain, it was estimated that there are over 510,000 *Phoenix* palms in an area of 5816 km^2^ (Culman et al. 2020).

Palm trees within the Euro-Mediterranean Region provide a variety of ecosystem services. According to de Groot et al. (2002), ecosystem services are services resulting from natural processes or functions that directly or indirectly fulfil human requirements (but also see Potschin and Haines-Young (2016)). Values for provisioning services (e.g. date production) in Europe were estimated to be worth around $16 million (Audsley and Charlton 2016). Further ecosystem services of palms exist, but defining a complete list and placing an economic value on these is challenging. Benefits include air purification, shade, reductions in ultraviolet radiation, production of oxygen and reduction of carbon dioxide, recreational opportunities and lower levels of noise and dust. A study in Barcelona, Spain, estimated that 1.4 million urban trees (including palms) removed 305.6 tonnes of air pollution (valued at €1 million) and sequestered carbon estimated at 5,422 tonnes/year (Baró et al. 2014). Cultural services of palms include ornamental palms in an urban environment, such as lining streets, parks, heritage palm groves and private gardens.

In addition, the ecosystem ‘palm tree(s)’ offers several ecosystem functions resulting in indirect services that can be summarised as habitat functions (de Groot et al. 2002). These include provisioning of habitat, food and shelter for a variety of animal species and “thereby contribut[ing] to the (in situ) conservation of biological and genetic diversity and evolutionary processes” (de Groot et al. 2002). As ornamental palms are often intensively managed, research interest in their ecological function has largely been conducted on palm species in their natural native range (e.g. the Canary Islands for *Phoenixcanariensis* (Cabrera et al. 1989, Meekijjaroenroj and Anstett 2003) or is strongly biased towards (intensively) managed date palm plantation and/or fauna species considered as pests (e.g. insect pests (Blumberg 2008, Al Antary et al. 2015) or non-arthropod pests (Alia et al. 2014, El-Shafie and Abdel-Banat 2018) on date palms). Few studies have looked at palm-animal interactions (Muñoz-Gallego et al. 2023) or the broader picture of animal communities and numbers of fauna species found (i.e. species richness). In managed palm groves and plantations in northern Africa and the Arabian Peninsula, the arthropod community in general (Deghiche-Diab et al. 2015) and on the soil surface (Mehdi and Mohamed 2015) and, more specifically, oribatids (Djelloul and Djamel-Eddine 2012), butterflies (Zeghti et al. 2019) and grasshoppers (Zergoun et al. 2018) have been described and partially compared with other habitats (Hadjoudj et al. 2018, Deghiche-Diab et al. 2020). For vertebrates, data exist from the same region on bird communities in palm groves (Guezoul et al. 2013) and on reptiles in different habitats including palm groves (Mouane et al. 2021). However, data are lacking for the Euro-Mediterranean Region for both invertebrates and vertebrates.

This study aimed to monitor the invertebrate and vertebrate fauna (hereon referred to as faunistic richness) occurring on and associated with ornamental palms of the genus *Phoenix*, over the course of one year. The term ‘ornamental palm’ in this context refers to the type of trees (i.e. non-native, planted palm trees) rather than their current use at the study sites. Five different locations and settings were chosen in southern Spain, as a representative Mediterranean region where ornamental palms are commonly used and that is known for its “palmerals” (palm groves) (Rivera et al. 2015). Four of these sites were chosen to be cultivated and in areas with low human disturbance, with varying levels of current management (see Suppl. material 1). For contrast, two mature trees were sampled at a fifth site containing six intensively-managed ornamental palms in an area with high human disturbance. In order to prepare an inventory as complete as possible, several complementary methods were chosen. However, not all methods could be applied at the fifth site due to restricted accessibility of the area and of the pruned trees and due to interference with the general public. Monitoring methods were applied monthly in order to assess the vertebrates and invertebrates associated with the full seasonal cycle of palms, including flowering and fruiting.

## Material and Methods

### Study Sites

Study sites were selected to cover the broadest range of fauna using ornamental palms, both as a temporary and permanent habitat. The study was conducted at four sites in south-eastern Spain: VE (‘**V**ivero **E**sther Alfonso Daimes’, near La Marina, Province of Valencia), AB (**Ab**anilla, Province of Murcia), LS (‘Huerto de **l**a **S**eca’) and CM (‘Huerto del **C**ura **M**oscardó’; both south of Elche near Matola, Province of Valencia). These sites consist of diverse, planted *Phoenix* sp., mainly *P.dactylifera* mixed with some *P.canariensis*, with trees of different ages and heights and including both male and female trees. Site VE was considered ‘coastal’, being situated ~ 7 km from the coast and, therefore, influenced by littoral conditions (e.g. salt spray, regular winds and more moderate temperatures). Site AB was located ‘inland’ and exhibited less wind, less humidity and a broader temperature range. Sites LS and CM were chosen to complement these first two sites through the structure and composition of the palm groups: LS included trees of different heights and ages, while CM was a traditional orchard with rows of palms located around crop fields. A fifth site, SP (**s**wimming **p**ool, Abanilla, Province of Murcia), consisting of two mature, intensively treated and managed trees located in a swimming pool complex, was included to compare faunistic richness in a more urban scenario.

The five sites varied in the amount of management, from none (VE), minimal (i.e. physical management like cutting dead leaves took place, but little or no chemical treatments were applied), to intensive (SP; i.e. pruning, irrigation, pesticide treatment; Suppl. material 1). Palms at all sites, except for SP, were allowed to flower and produce fruits that were not commercially harvested. For more details of the selected sites and palm trees, see Suppl. material 1.

### Sampling Methodology

Palm trees were surveyed for over 3,000 hours during the course of one year from October 2020 until September 2021. Several complementary methods were chosen in order to prepare an inventory as complete as possible for invertebrates and vertebrates using the palm trees. Thus, the sampling methodology was designed to only record observations on the trees and their immediate surroundings, i.e. the perimeter of the tree including the crown, trunk and ground underneath, as well as the air space in between. Not all methods could be applied at site SP due to restricted accessibility to the swimming pool complex (i.e. no access outside staffed hours, no photo or video recordings possible, no permanent installation of expensive equipment). In addition, the monitoring coincided with the Covid-19 pandemic and its working restrictions in Spain.

#### Invertebrates

The following invertebrate sampling methods were selected to collect flying arthropods (e.g. Hymenoptera, Diptera, Lepidoptera, Coleoptera, Hemiptera), surface-dwelling arthropods (e.g. Coleoptera, Araneae and Acari) and pollinators at the time of flowering (i.e. from opening of the first buds until withering of the last flowers). All invertebrates were recorded. Both flowering and non-flowering palms were selected during sampling sessions. For details, see Table 1. For example photographs of some methods, see Suppl. material 2.

**Air eclectors** (‘air eclector according to Rahn’, Bioform) were placed in the crowns of male and female palm trees at a height of at least 3 m. Each air eclector included two trapping vessels, one at the top and one at the bottom of the collection area. The collection area of the eclector was white in order to minimise the possibility of either attracting or repelling invertebrates (though either possibility cannot be completely excluded). A saturated sodium-chloride solution with a small amount of copper sulphate for conservation was used for fixation of the animals in the traps.

**Trunk eclectors** were placed around the trunks of male and female palm trees, at heights of approximately 1 - 4 m. They consisted of tarp wrapped around the trunk fixed with ropes (e.g. Basset et al. (1997)). Due to the uneven structure of the palm trunks, holes between the trunk and the eclector were filled with sealing mass to prevent invertebrates from bypassing the eclector. Invertebrates were collected in foldings of the tarp. For sampling, the tarp was carefully opened and shaken into an entomological conical umbrella.

**Beating and manual collection** from leaves, inflorescences, infructescences and trunks. Invertebrates were collected manually with tweezers or beaten down into a tray/funnel by shaking leaves, inflorescences and infructescences according to Basset et al. (1997).

An **aspirator (D-Vac Suction Sampler)** (STIHL, for example, Motamedinia and Rakhshani (2017)) equipped with combustion engine and a suction tube with a sampling bag was used to collect invertebrates located on or flying close to palm trees. Samples were taken from near the crowns and on the trunks. At site SP, samples could only be taken from the trunk.

**Leaf washing** after leaflets were removed from palm trees, cut into smaller pieces if needed and washed to collect mites and other invertebrates attached to the leaves (method according to Boller (1984), Jesse et al. (2006)). Leaflets were left to soak in a water/detergent solution (one drop of liquid washing detergent per litre tap water) for at least one hour. Subsequently, single leaflets or leaflet pieces were removed and washed with tap water over a funnel with a fine sieve. This procedure was repeated for every single leaflet/piece. The remaining liquid from the vessel was also poured through the funnel into the sieve. Afterwards, the vessel was rinsed with tap water and the content was poured through the sieve as well. The content of the sieve was separately flushed with ethanol into pre-labelled sample vessels.

**Flower/fruit washing** analogous to the leaf washing method was undertaken when flowers and/or fruits were present (from December 2020 onwards).

**Observations** of invertebrates on palm trees during vertebrate surveys (i.e. visual and acoustic observations, artificial refuges, palm tree inspections, wildlife cameras; see below) were recorded. When it was not possible to count the exact number of individuals, the number of individuals/observations was set to 100 (ants only), 50, 25 or 15.

**Storage and Identification**

All samples were stored in 70% ethanol in the dark at ambient temperatures. The samples collected in April 2021 from the air eclector, trunk eclector, beating and manual collection and aspirator (D-Vac) sampling methods were lost during shipment.

Invertebrates were individually identified to family level using dissecting microscopes and counted. Identification keys were used when necessary: Gisin (1960), Chu and Cutkomp (1992), Karg (1993), Mason and Huber (1993), Pérez-Iñigo (1993), Arnett Jr. et al. (2002), Weigmann (2006), Krantz and Walter (2009), Bellmann (2016), Nentwing et al. (2017), Schaefer (2018) Anonymous (2021). Taxonomy was adapted according to: GBIF.org (2021) and eu-nomen.org - PESI (2023).

#### Vertebrates

The following sampling methods were selected to record vertebrate species present. For details, see Table 1. For example photographs of some methods, see Suppl. material 2.

**Visual and acoustic observations** of animals seen or heard in/on/around palm trees (species, number and their behaviour) were recorded by one observer. Observations were done for two hours in the morning (shortly after dawn), two hours in the evening (shortly before dusk) and two hours at night (shortly before/after twilight; using night vision devices, infrared cameras and/or spotlights). For each site, pre-defined routes between or close to the palm trees were used for observations (adaptation of bird point count method following Fuller and Langslow (1984)).

A **Bat detector** (Echo Meter Touch 2 bat detector for Android, Wildlife Accoustics) was used for recording and identifying bat species during the night-time visual and acoustic observations. Bats could be detected within about a 10 m radius from the observer.

**Palm tree inspections** including the ground around the trunk(s), the trunk(s) themselves and the crown(s) were made for signs of animal use (e.g. burrow entrances, cavities used for roosting or breeding, faeces, food remains).

**Wildlife cameras** were installed on the trunks of different palm trees at heights of 1 - 5 m to record animal activity during the day and at night. Cameras were focused on crowns, trunks and bases of palm trees. Cameras were checked monthly to download the data and replace the batteries if needed. Photos were analysed and all animals detected were recorded. For multiple pictures of animals highly likely to be the same animal, only the first picture was registered each day. Wildlife cameras were set up and started recording on the 29/09/2020 at sites VE and LS. Observations made during these two days were included in the month of October (2020) as the month of September was fully recorded the following year (2021). This resulted in eight additional observations, all of which were mammals.

Two kinds of **track tubes** were used. First, Black Trakka monitoring tunnels (100 mm x 100 mm x 500 mm, Gotcha Traps Ltd.) were placed in crowns and on the base of trunks of up to five palm trees at each site. Second, tubes made of thin plastic boards (trunk tunnels) were fixed horizontally on trunks of up to five different palm trees at each site. Both tubes were lined with inked Black Trakka monitoring cards (Gotcha Traps Ltd.). The tubes were checked at least once per month. Identification was done, based on expert knowledge and the following references: Sanz (1997), Abenza (2018), Sanz Navarro and Turón Gil (2018), ZooBot (2023). Tracks were only used if they could be identified to the appropriate taxonomic level (family level for invertebrates and species level for vertebrates).

**Small mammal traps** (Ugglan traps, Grahnab) were placed around the base of trunks, on trunks and in the crowns of palm trees. Traps were baited with wheat seeds, activated in the evening and checked the following morning. For animal welfare reasons, traps included a small escape hole for shrews.

**Artificial refuges for reptiles** (arboreal and terrestrial refuges) were installed on tree trunks and on the ground between palm trees. Arboreal refuges consisted of squares of synthetic fibre mats (size 1 m x 1 m), placed around the trunk and tied with a string (following Michael et al. (2018)). Terrestrial refuges consisted of squares of roofing cardboard (size 0.5 m x 0.5 m), placed on the ground and fixed in corners, for example, with stones (following Vlašín and Mikátová (2015)). The terrestrial refuges served as controls to distinguish between reptile species seeking shelter in general and those more likely to be using the palm trees as a specific habitat. During palm tree inspections, the refuges were checked by lifting the mats to record all reptile species underneath.

Vertebrates were determined to species level whenever possible.

### Data Evaluation

Records of individuals not identified to family level for invertebrates or species level for vertebrates were excluded from data evaluation. However, such records were considered an additional family/species when no other families/species were recorded within the next higher taxon. For example, this resulted in Astigmata, Symphypleona and Embioptera being considered invertebrate 'families'. Due to differences in sampling effort, records of the site SP were only used for site comparisons for invertebrates and excluded from all other analyses.

For invertebrates, the abundance measures reported represent combined numbers of individuals captured and observed. For vertebrates, the abundance measures reported represent the numbers of observations and, therefore, may not be equivalent to the numbers of individuals present.

Richness (as total number of families for invertebrates and total number of species for vertebrates) was evaluated according to ecological requirements and ecological niche, respectively. Consequently, the focus was more on diet for invertebrates (rather than taxonomic group) and more on taxonomic class for vertebrates.

Invertebrates can display wide variability in terms of diet within family level. Expert opinion was used to determine the diet of an invertebrate family in terms of how *Phoenix* palm trees were used as a food source by that specific family. For families displaying a variety of diets, the direct ingestion of plant tissue (e.g. sap or leaf tissue) was prioritised over indirect ingestions (e.g. detrius material or prey). Therefore, the diet assigned to a family level (hereafter referred to as 'palm-associated diet', assumed, based on judgement) aims to describe the maximum potential value provided by *Phoenix* palm trees in the Mediterranean Region. Families were divided into the following 'palm-associated diet' guilds. Predatory as well as parasitic groups were classified as ‘predaceous’. Families which directly feed on plants by either piercing into plant tissue or chewing on plant tissue were termed ‘herbivorous’, families feeding on dead wood ‘sapro-xylophagus’. Flower-visiting families potentially feeding on pollen or nectar were termed ‘palynivorous/nectarivorous’. Primary and secondary decomposers were termed ‘detritivorous’. Families predominantly feeding on fungi were termed ‘fungivorous’. Families which include mainly generalist feeders were termed ‘omnivorous’.

## Results

The raw data were published through GBIF: https://doi.org/10.15468/yj5c5j.

### Invertebrates

Samplings resulted in a total of > 105,000 collected individuals and > 12,000 observations. Data of individuals that could not be determined to family level were excluded from analysis. Similarly, due to differences in sampling effort, data collected at site SP were excluded from analysis and included only for site comparisons. This reduced the dataset to > 40,000 sampled individuals/observations belonging to 215 different families across seven classes: Insecta, Arachnida, Entognatha, Gastropoda, Malacostraca, Diplopoda and Chilopoda. Most individuals sampled belonged to the Acari, which made up more than half of the invertebrates found on the palm trees. Two other prevalent groups were the Psocoptera and the Collembola which were recorded by all sampling methods. Other families frequently found in relatively high abundances at all sites and throughout the year included the Curculionidae, Latridiidae, Tettigoniidae, Formicidae, Dictynidae, Philodromidae, Salticidae, Theridiidae and Helicidae. Infrequent findings at low numbers comprised families which are usually very common, but do not depend on palm trees, like Empusidae, Mantidae and various families of the Odonata. Other families like Buprestidae and Cerambycidae may contain very rare and potentially endangered species, but also pests that directly depend on palm trees for their larval development. A full list of the taxa identified is given in Suppl. material 3 and data on their abundances in Suppl. material 4.

**Figure 1.**
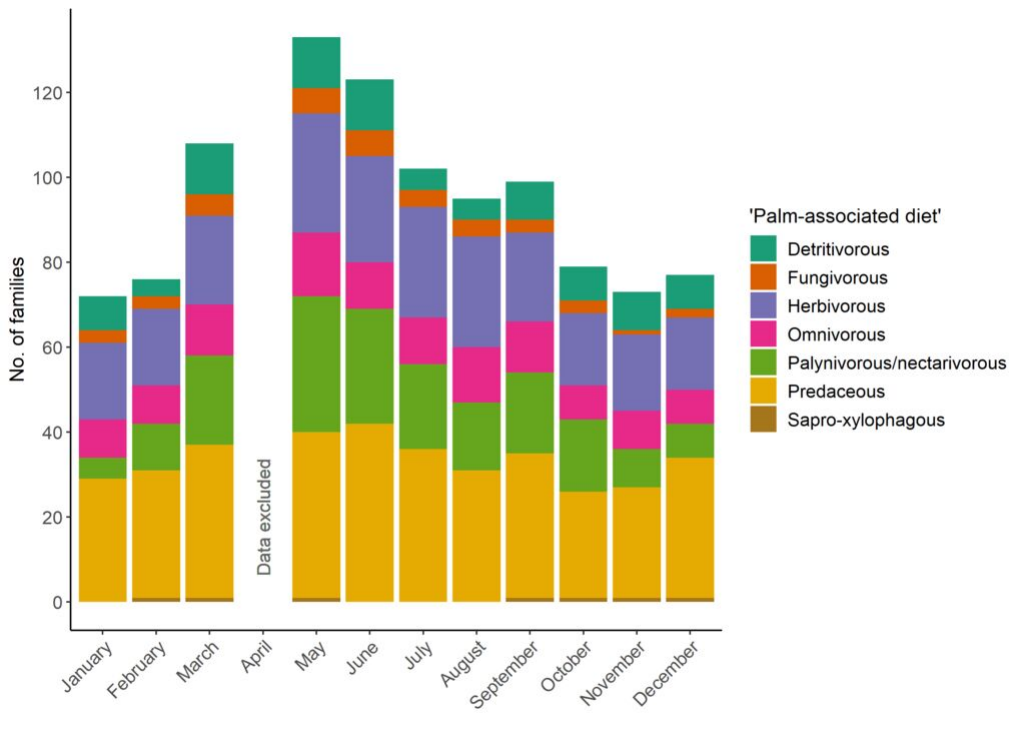
Number of invertebrate families recorded per 'palm-associated diet' guild and month. Site SP was excluded because not all methods could be applied at site SP due to restricted accessibility to the swimming pool complex (i.e. no access outside staffed hours, no photo or video recordings possible, no permanent installation of expensive equipment). As some April samples were lost during shipment and, hence, the data are not comparable with other months, April was excluded.

**Figure 2.**
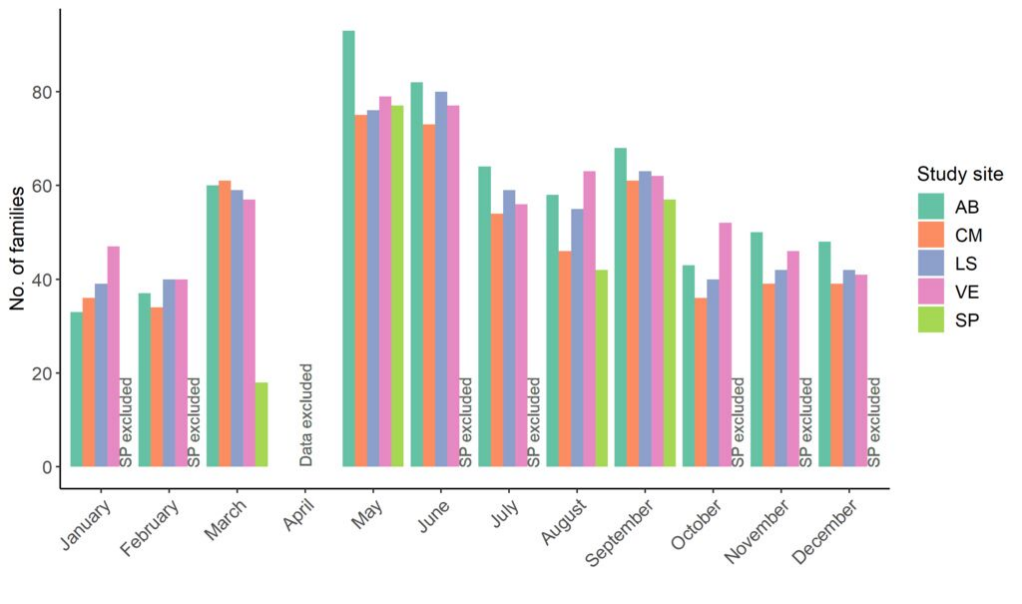
Number of invertebrate families recorded per site and month. As some April samples were lost during shipment and, hence, the data are not comparable with other months, April was excluded. Data of site SP are only shown when the effort at that site was comparable to the effort at the other sites.

The richness (i.e. number of families) was highest in late spring and summer, with a maximum of 133 of the 215 families found in May; and lower in autumn and winter, with a minimum of 72 families in January (Table 2). Somewhat different families were found over the course of the year. The most diversely composed 'palm-associated diet' guild was the group of predaceous invertebrates, followed by the herbivorous and, in some months, the palynivorous/nectarivorous groups (Fig. 1); only a few sapro-xylophagous and fungivorous families were observed. However, a group with many families was not always a group with many individuals, for example, the palynivorous/nectarivorous group was one of the groups with fewer individuals recorded (see Table 2). Despite the selection of different and somewhat complementary sites, the richness was fairly similar between AB, CM, LS and VE (Table 3, Fig. 2). Months with overall higher richness were of higher richness in all sites and vice versa. Additionally, most families were recorded in all four fully-sampled sites. In site SP, data comparable with the other sites could only be collected in March, May, August and September, while several methods needed to be omitted in the other months due to restrictions at the site. Apart from March, the richness in SP was as high as in the other sites. For a distribution of the families across sites and classes, see Table 4.

The richness, as well as the number of individuals, was highest in the crown of palm trees at any time of the year and lowest in the air and on the base around the trees. However, differences in the selectivity of the methods and the sampling effort cannot be accounted for.

In addition to the above-mentioned methods, casual observations were also recorded whenever a new invertebrate family was found during fieldwork (i.e. observed outside of sampling methods mentioned in the Methods section), which mainly included larger individuals visible to the naked eye. This resulted in one additional family (Pterophoridae). This observation is not included in any of the summary tables or figures.

### Vertebrates

A total of 7,894 observations, of 87 species, were recorded throughout the course of the study. Observations consisted mostly of bird and mammal species (34.9% and 64.4%, respectively). The number of observations per species varied widely, from 1 to a maximum of 596 for birds (blackbird, *Turdusmerula*), a maximum of 1,989 for mammals (black rat, *Rattusrattus*) and a maximum of 29 for reptiles (Algerian sand racer, *Psammodromusalgirus*). A full list of the taxa observed is given in **Appendix 1**.

**Figure 3.**
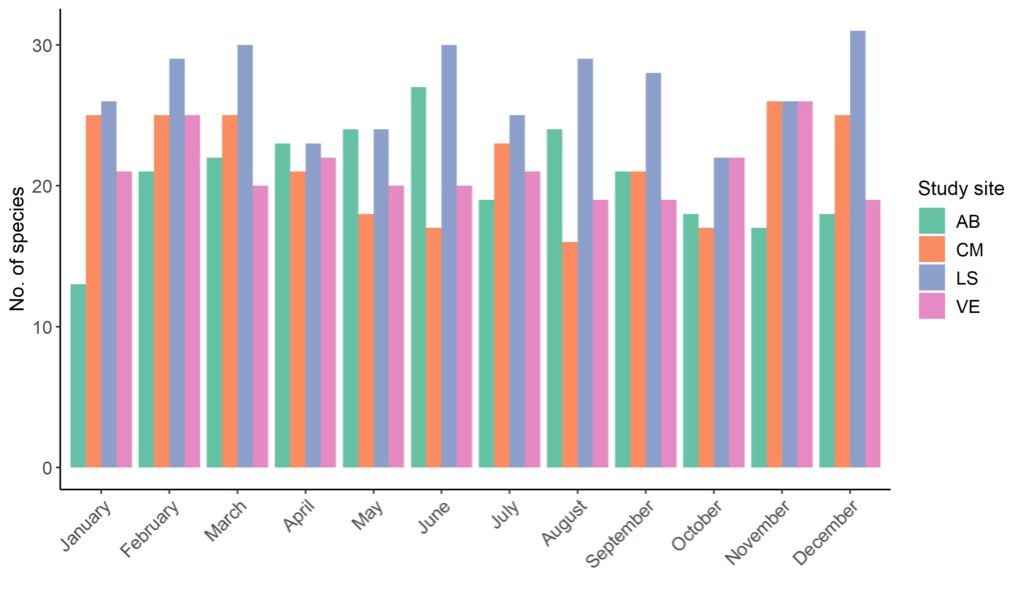
Number of vertebrate species observations recorded per site and month. Site SP was excluded because not all methods could be applied at site SP due to restricted accessibility to the swimming pool complex (i.e. no access outside staffed hours, no photo or video recordings possible, no permanent installation of expensive equipment).

Overall the number of species was highest in November, with a maximum of 50 species observed and lowest in October with a minimum of 36 species observed (see Table 5, Fig. 3). There was little variation in species richness between sites (Table 6).

**Figure 4.**
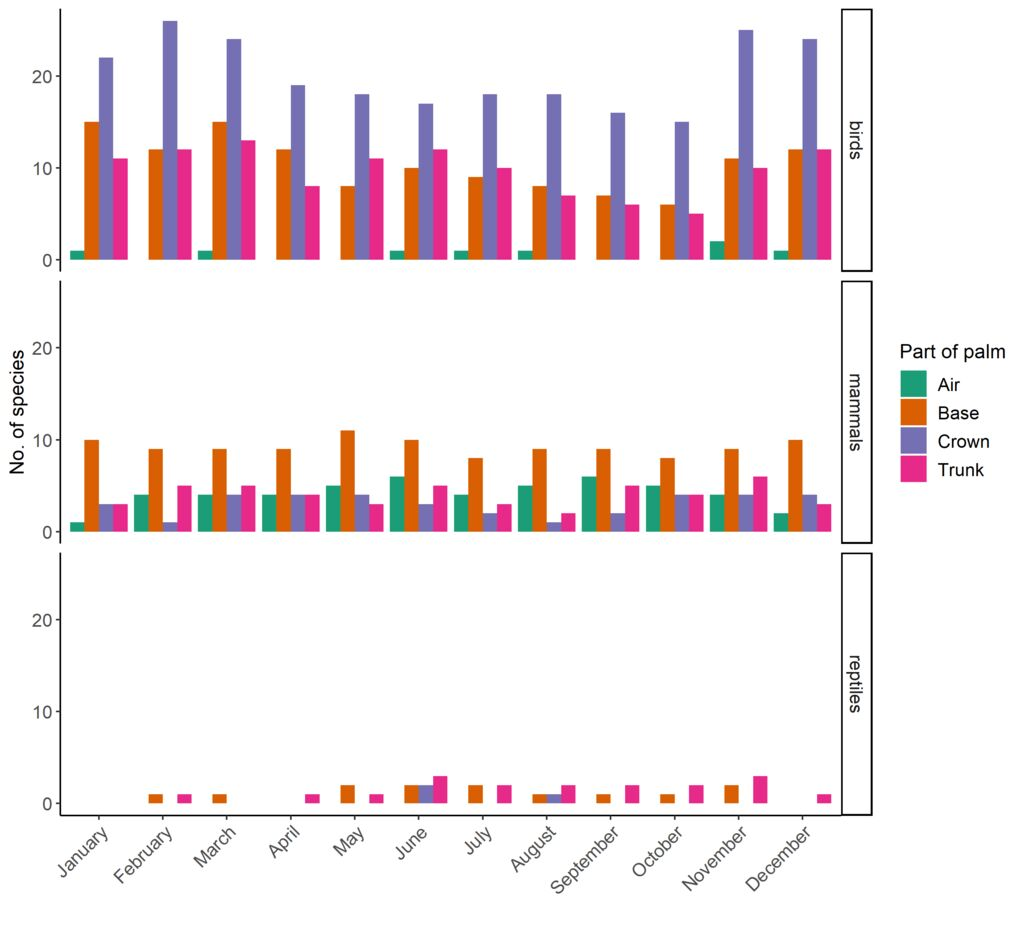
Number of bird, mammal and reptile species observed, per month, on different parts of palm trees across all sites. Site SP was excluded because not all methods could be applied at site SP due to restricted accessibility to the swimming pool complex (i.e. no access outside staffed hours, no photo or video recordings possible, no permanent installation of expensive equipment).

For birds, 61 species were recorded, eight of which were recorded every month. In addition, the redwing (*Turdusiliacus*) was observed as a 'casual observation’ (i.e. outside of actual sampling time) in November 2020 at the base of a palm tree at study site AB. This observation is not included in the summary tables or figures. The number of species observed was highest in late autumn, winter and early spring (November, January-March) and lowest in early autumn (September-October; Table 5). Species richness was highest in the crown of the tree and lowest in the air surrounding the tree. Only 11 observations were made of bird species using the air surrounding the tree throughout the study (Fig. 4).

For mammals, 20 species were recorded, seven of which were recorded every month. Species richness was highest in late spring and early summer (May-June) and lowest in winter (December-January), but also in July (Table 5). Overall, species richness was highest at the base of the tree and lowest in the crown (Fig. 4). Only bats were observed in the air surrounding the palm trees and, in total, seven species were recorded.

For reptiles, five species were recorded. No reptile species were recorded consistently across months. Species richness was highest in summer, but also in November and lowest in winter (Table 5, Fig. 4). Only one carnivorous species was observed (horseshoe whip snake, *Hemorrhoishippocrepis*), at the base, on the trunk and in the crown of palm trees (i.e. at all height levels). All other species observed were insectivorous. In addition, the spiny-footed lizard (*Acanthodactyluserythrurus*) was observed as a 'casual observation’ (i.e. observed outside of sampling methods mentioned in the Methods section) in October 2020 at the base of a palm tree at study site AB.

Only one single amphibian species was recorded during monitoring (Perez's frog, *Pelophylaxperezi*), in May at the base of a tree at site AB.

## Discussion

The aim of this study was to record the fauna associated with ornamental palm trees (i.e. non-native type, planted trees) in the Euro-Mediterranean area, thereby closing the existing data gap between so-called pest species in non-European date palm plantations, palm species in their native range and surveys restricted to specific taxonomic groups. The study was run in Spain for one year, from October 2020 to September 2021. The study was largely successful, despite being hampered by the unforeseen Covid-19 pandemic. There were > 3,000 hours of observations/samplings in the field, resulting in ~ 7,900 observations of vertebrate species and > 100,000 invertebrates counted in the laboratory from the field samples (with > 40,000 being identified to family level).

The complementary set of methods used resulted in the identification of 216 invertebrate families from seven different classes and 89 vertebrate species, consisting of 62 birds, 20 mammals (including bats), six reptiles and one amphibian species associated with *Phoenix* palms. Several authors have investigated richness of fauna in date palm rich habitats in Algeria and found 69 arthropod families in an oasis (Deghiche-Diab et al. 2015) and in palm groves 14 and 20 arthropod families (Mehdi and Mohamed 2015, Hadjoudj et al. 2018), 13 Lepidoptera families (Zeghti et al. 2019) and four grasshopper families (Zergoun et al. 2018). Similarly, vertebrate assessments resulted in 59 bird (Guezoul et al. 2013) and 30 reptile species (Mouane et al. 2021). Even if such comparisons to the current study should be made with caution due to differences in location, methods and, most importantly, due to restrictions of focusing on specific taxonomic groups, it shows that *Phoenix* palms provide a habitat for many species and individuals in the course of one year.

In the present study, similar to previous studies by Castro-Caro et al. (2014) and Zeghti et al. (2019), the composition of invertebrate families and vertebrate species exhibited changes throughout the year, although these changes varied across different taxonomic groups (see Results). Generally, the number of invertebrate families was highest during the spring and summer months and strongly associated with the seasonal conditions and foliage availability needed for activity, food and reproduction. However, the abundances and densities decreased in the hottest months, indicating suboptimal conditions, such as high temperatures and drought and possibly a lack of shelter in the dry ground vegetation. Such a decrease was previously described for butterflies (Zeghti et al. 2019), but likely also occurs in other taxonomic groups. Independent from these seasonal changes, faunistic richness was highest in the predaceous group, mainly caused by a high richness in predaceous spiders (Araneae). This is not surprising given that spider diversity increases with more diverse and structured habitats (Jiménez-Valverde and Lobo 2007, Damptey et al. 2022), which may also be true for other, non-Araneae predators (Aguilera et al. 2020). Similarly, the palynivorous/nectarivorous group demonstrated high family richness during most of the year, despite *Phoenix* species flowering for only a short period of time during the year and being predominantly wind-pollinated (but see Sosa et al. (2021)). This group mostly included families other than the ‘classic’ pollinators and were mainly those that lack a collecting apparatus (e.g. as expressed in *Apismellifera* (Apidae)) and/or do not have the body size or the mobility to transport pollen between flowers. However, specific relationships between palms and small ‘non-classic’ herbivorous pollinators, such as *Neoderelomuspiriformis* beetles, have evolved, with the trees providing food for adults and larvae in exchange for pollen deposition (Meekijjaroenroj and Anstett 2003) and such relationships may also exist for other families (Sosa et al. 2021). Overall, the high faunistic richness demonstrates the importance of pollen and flowers as a food source, but also of the inflorescence itself as habitat for small arthropods.

Some commonly-known pest species of palm trees were observed, but in very low numbers (e.g. *Rhynchophorusferrugineus* (four individuals) or *Paysandisiaarchon* (two individuals)). Families containing also less specialised pest species (i.e. pests of not just palm trees) were observed more frequently (e.g. other Curculionidae, Nitidulidae, Aphididae, Aleyrodidae). Overall, our results show the diversity of invertebrates which can be supported by *Phoenix* palm trees even at family level. However, as invertebrates were usually not determined to species level, no assumptions or conclusions regarding species can be drawn.

For birds, the most frequently observed species were blackbirds (*Turdusmerula*, 596 observations), blackcaps (*Sylviaatricapilla*, 289 observations), robins (*Erithacusrubecula*, 196 observations), great tits (*Parusmajor*, 172 observations) and little owls (*Athenenoctua*, 165 observations). Twenty-five species (~ 40% of total) were recorded fewer than five times throughout the study. The number of species observed was highest in the colder periods and lowest in early autumn (Table 5; Fig. 4). Generally, the Mediterranean area is an important over-wintering habitat for many birds migrating from central and northern Europe to the Iberian Peninsula (Moreau 1956). Palm trees are a potential food source by providing energy-rich fruit (see Spennemann (2019) for details for *P.canariensis* drupes) to frugivorous migratory species and invertebrates to insectivorous and omnivorous species. Dates were present on trees throughout the year, except for July-September and present in highest concentrations in November-February, matching the peak in bird abundance observed in this study, as well as the highest peak of frugivorous bird abundance in the Mediterranean Basin (Rey 2011 and references therein). Fruits from *P.canariensis* have been shown to provide a stable food source for several bird species in Australia (Spennemann 2019), but little research has been done in the Mediterranean. On the Canary Islands, fruits of *P.canariensis* have been documented to be taken by blackbirds (Sosa et al. 2021) and common ravens (*Corvuscorax*, Nogales et al. (1999)). In this study, 12 bird species were spotted feeding on dates, at least three of them being migratory (blackcap *Sylviaatricapilla*, chiffchaff *Phylloscopuscollibita* and common starling *Sturnusvulgaris*). Several bird species are known to nest in *Phoenix* palms (see El-Shafie and Abdel-Banat (2018) for examples for date palms), both in their native habitat (e.g. rose-ringed parakeets *Psittaculakrameri* in *P.canariensis* in Tenerife (Hernández-Brito et al. 2022)) and in mainland Spain (e.g. monk parakeet *Myiopsittamonachus* mostly in *P.canariensis* (Molina et al. 2016)). In the current study, observations were made of three species gathering nesting material (mistle thrush *Turdusviscivorous*, blackbird and serin *Serinusserinus*), an active blackbird nest and a jackdaw (*Coleusmonedula*) inside a hole in a dead palm tree trunk.

For terrestrial mammal species (i.e. non-bat species), the differences in species richness between months can likely be explained by seasonal differences. Furthermore, many of the species are associated with humans (e.g. the domestic dog, domestic cat) or commonly found in different landscape types, being ubiquists. Ripe fruits were an important food source for some mammals (e.g. brown rats). The most frequently observed mammals were rats (*Rattusrattus*, 1989 observations), European rabbits (*Oryctolaguscuniculus*, 1361 observations), red fox (*Vulpesvulpes*, 481 observations) and domestic cats (*Feliscatus*, 445 observations). The least frequently observed mammals were greater white-toothed shrews (*Crocidurarussula*, three observations), savi’s pipistrelles (*Hypsugosavii*, one observation), Schreiber's bats (*Miniopterusschreibersii*, five observations) and European free-tailed bats (*Tadaridateniotis*, two observations). For bats (seven species in total, all insectivorous), the highest species richness correlated with months when insect numbers were also highest. In Israel, bat species richness and activity was found to be highest during summer date harvesting, potentially because of increased insect activity (Schäckermann et al. 2022). In the current study, January and December had the lowest bat species richness (one and two species, respectively). All observed bat species hibernate in winter, but will experience torpor breaks if climatic conditions are favourable (e.g. Barros et al. (2021) and references therein). Screiber's bat, a cave-dwelling species listed as "vulnerable" in Europe (Cistrone et al. 2023), was observed five times throughout the survey (once each in May, June, September, October and November), at three of the sites. Regionally (Murcia Province, Spain), this species has declined by 70% in the last decade (Lisón et al. 2011).

Few reptile and amphibian species were recorded in this study. The Mediterranean house gecko (*Hemidactylusturcicus*), the Algerian sand racer (*Psammodromusalgirus*) and the common wall gecko (*Tarentolamauritanica*) were observed similarly frequently, all usually on the tree trunk, showing that these species utilised palm trees. No literature is available on how much, if at all, fruits from palm trees are part of the diet of reptiles in the Mediterranean Region; however, seeds of *P.canariensis* have been found in the faeces of island-endemic *Gallotia* lizards on the Canary Islands (Valido Amador 1999). The ocellated lizard (*Timonlepidus*), a species listed as "near threathened" in Europe (Pleguezuelos et al. 2009), was observed twice at site AB (once in May and once in June).

The four main study sites were very similar in terms of faunistic richness; however, a broader range of sites might reveal differences in the family/species composition and this merits further investigation. Overall, denser vegetation may be a better habitat for flightless invertebrates, while more open habitats may be better for colonisation by flight (Peng et al. 2020, Nardi et al. 2022). This could be similar for vertebrates, with birds (and bats) preferring more open habitats, but the study sites were most likely too few to detect such effects within the investigated sites in Spain. Surprisingly, despite reduced area investigated and sampling effort, the invertebrate richness at site SP was similar to the other sites for the months in which sampling effort was comparable. Only in March, the number of families observed was lower than at all other sites and likely due to tree management executed in the preceding weeks. This generally suggests that small patches (i.e. single trees) are sufficient to serve as suitable habitat, while larger palm ecosystems may additionally act as a source for emigration, especially for invertebrates. As the study sites and methods were chosen to complement each other in terms of tree density, age, structure, height and management practices, in order to compare between sites, an increased number of diverse sites (i.e. ranging from totally unmanaged to commercial plantations to ornamental trees) and a greater sampling effort would be required to retrieve more detailed data on biodiversity and potential differences. Furthermore, identification to a lower taxonomic level and a better understanding of biotic and abiotic factors would be beneficial. Other studies have, for example, investigated the relationship of richness or species composition to the age of a plantation and found mixed results (Dislich et al. 2017). To further investigate the impact of management, samplings before and after certain management measures or a more experimental approach would be needed.

Despite the careful selection of complementary methods and sites, there were some weaknesses in this study. Certain methods used were designed to be general (e.g. observations), while others were more selective for certain taxonomic groups or certain parts of the palm trees. For example, small mammal traps are aimed specifically to trap mice and voles, but exclude small shrews (for animal welfare reasons, the traps have an escape hole to prevent them from starving in the traps due to their fast metabolism). Likewise, trunk eclectors are specific for trapping invertebrates crawling along the trunk, but exclude invertebrates using other parts of the palm trees. In addition, no organisms using the root system were sampled. Furthermore, some methods could only be used to record numbers of observations rather than individuals (because individuals were not uniquely marked), while others resulted in discrete numbers of individuals. Finally, all methods only covered one year and a monthly ‘sampling’ only represents a snapshot within each month. Thus, the data may not accurately represent all fauna that were present and using palm trees and potential irregularities of the studied year cannot be separated from other effects/variables, nor may be an accurate description of faunistic richness of that specific month. Nevertheless, the results show the minimum faunistic richness present in ornamental palms in the studied area in Spain and indicate diverse invertebrate and vertebrate communities associated with ornamental palm trees in the course of a year.

While the results show that some extent of management does not deter wildlife, the ecosystem value for biodiversity in palm groves can be improved with little effort. Inflorescences that are usually cut to reduce dirt and litter of palms not serving to produce dates (Hodel 2009) could be left to provide food and habitat for animals, also later in the season when turning into sugar-rich fruits. Management activities could be reduced to the absolute necessary, understorey vegetation left to provide additional habitats to hide and to emigrate from into the palm trees (Azhar et al. 2011) and old and/or unused palm groves and trees preserved. Similarly, trees of different age categories could be included in palm groves for a higher habitat diversity (Azhar et al. 2011). A summary of strategies for increasing the ecological value of palm oil plantations, which can also be applied to other palm groves, can be found in Dislich et al. (2017) (Table 3 and section 8 thereof). However, these strategies may cause conflicts with other ecosystem functions (Bryan et al. 2010, Setälä et al. 2014, Schirpke et al. 2020), especially in more populated or urban areas where palm trees are used for recreational, cultural or touristic purposes (e.g. in the Elche area in Spain). Conflicts can arise from various sources, such as dirt coming directly from flowers and fruits or indirectly from animals eating those fruits. Noise from animals living in the palm trees (e.g. parakeets, Mori et al. (2020)), insects that can sting people, allergic reactions to pollen or the untidy look of palm trees and groves when not heavily managed (e.g. by old leaves, dense or dry understorey vegetation) can all contribute to conflicts. Finally, conflicts may occur by taking space that could otherwise be used for leisure activities, houses or crops (Setälä et al. 2014). An evaluation of cultural ecosystem services provided by palm trees in the Euro-Mediterranean area should be performed to explore conflicts and limitations in order to address land management and decision-making processes, similar to what has been done in mountain settings (Schirpke et al. 2020).

## Supplementary Material

0572C569-DF42-5158-B788-C8D657B37EB7 10.3897/BDJ.12.e123144.suppl1 Supplementary material 1 Description of Study Sites Data type additional information Brief description Descriptive information on dimensions of sites, palm trees, undergrowth, topographical setting, soil surface, temporary water and management practices. File: oo_967655.pdf https://binary.pensoft.net/file/967655 Silke Laucht, Kaat Brulez, Jörg Hanisch, Alexander Blakey, Gabe Weyman, Jan-Dieter Ludwigs, Tania Alvarez

EAB2B21E-0F54-5437-9642-B87DE74FAFCC 10.3897/BDJ.12.e123144.suppl2 Supplementary material 2 Example photographs of some of the methods Data type photos File: oo_942278.pdf https://binary.pensoft.net/file/942278 Silke Laucht, Kaat Brulez, Jörg Hanisch, Alexander Blakey, Gabe Weyman, Jan-Dieter Ludwigs, Tania Alvarez

BC9F8229-1737-5DBB-AE90-455584793676 10.3897/BDJ.12.e123144.suppl3 Supplementary material 3 Complete list of families/species found associated with ornamental palm trees in Spain Data type occurrences of invertebrate families and vertebrate species Brief description Note: Taxonomic classification is ever-changing and differences may appear between sources. The following sources have been used here: https://www.gbif.org and http://www.eu-nomen.eu/portal/index.php. File: oo_969222.xlsx https://binary.pensoft.net/file/969222 Silke Laucht, Kaat Brulez, Jörg Hanisch, Alexander Blakey, Gabe Weyman, Jan-Dieter Ludwigs, Tania Alvarez

034E702D-C1BC-529E-8731-9982276F156F 10.3897/BDJ.12.e123144.suppl4 Supplementary material 4 Number of individuals per invertebrate family and month Data type occurrences Brief description Note: Site SP was excluded because not all methods could be applied at site SP due to restricted accessibility to the swimming pool complex (i.e. no access outside staffed hours, no photo or video recordings possible, no permanent installation of expensive equipment). As some April samples were lost during shipment and, hence, the data are not comparable with other months, April was excluded. File: oo_969864.xlsx https://binary.pensoft.net/file/969864 Silke Laucht, Kaat Brulez, Jörg Hanisch, Alexander Blakey, Gabe Weyman, Jan-Dieter Ludwigs, Tania Alvarez

## Figures and Tables

**Table 1.** Overview of methods used for monitoring. x = method suitable for this taxonomic group; - = method not suitable *activation in November 2020, first sample in December 2020.

**Method**	**Frequency**	**Part of the palm monitored**	**Duration**	**Birds**	**Mammals**	**Small** **mammals**	**Bats**	**Reptiles**	**Pollinators**	**Invertebrates**
**Visual and acoustic observations**	once a month	air, base, trunk, crown, leaves, inflorescences, infructescences	2 h mornings, 2 h evenings, 2 h night; not at site SP	x	x	x	x	x	x	x
**Palm tree inspections**	once a month	base, trunk, crown, leaves, inflorescences, infructescences	1-4 h together with artificial refuges; site SP: 12 to 38 min	x	x	x	x	x	x	x
**Wildlife cameras**	permanent	parts of trunk or crown	5 cameras per site; photos triggered by motion detection; not at site SP	x	x	x	-	-	x	x
**Track tubes**	permanently installed, check once a month	base, trunk, crown	whenever an animal walked through; 5 monitoring tunnels + 5 trunk tunnels per site; not at site SP	x	-	x	-	x	-	x
**Small mammal traps**	once a month	base, trunk, crown	one night; 30 traps per site; not at site SP; started mid-Nov. 2020	-	-	x	-	-	-	-
**Artificial refuges**	permanent, check once a month	trunk, surrounding ground	1-4 h together with palm tree inspections; 5 arboreal refuges + 15 terrestrial refuges per site; not at site SP	-	-	-	-	x	-	x
**Air eclectors**	12 to 18 days active; in Oct 20 active for 4-7 days	crown	4 per site; 1 at site SP in Dec 20, Jan 21, Mar 21 – Sep 21	-	-	-	-	-	x	x
**Trunk eclectors**	constantly from Nov 20 onwards; in Oct 20 active for 2-7 days	trunk	2 per site, checked 1-2x per month; at site SP from Nov 20^*^ onwards	-	-	-	-	-	-	x
**Beating and manual collection**	once a month	crown, leaves	5-15 leaves from 3-10 palms per site + ≤ 10 inflorescences (male + female) + ≤ 10 infructescences, 3 samples, each sampled for 4-6 min; not at site SP	-	-	-	-	-	x	x
**Aspirator (Dvac Sampling)**	once a month	crown, trunk	4-6 palms per site, each sampled for 230 - *c* 300 sec; at site SP: 1 palm, duration 120 sec, only in Mar 21 – May 21, Aug 21, Sep 21	-	-	-	-	-	x	x
**Leaf washing**	once a month	leaves	3 samples of 1-2 trees per site; per sample 10-20 leaflets depending on size; only 1 sample in Jan 21 at site SP	-	-	-	-	-	-	x
**Flower/fruit washing**	once a month, when present	inflorescences, infructescences	1-2 trees per site at least 1 inflorescence/ infructescence per site; 3 sites sampled per month, sites rotated, from Dec 20 onwards; not at site SP	-	-	-	-	-	x	x

**Table 2.** Number of invertebrate families (and individuals) per 'palm-associated diet' guild and month. Site SP was excluded because not all methods could be applied at site SP due to restricted accessibility to the swimming pool complex (i.e. no access outside staffed hours, no photo or video recordings possible, no permanent installation of expensive equipment). - = no specimens were recorded in that group. * one additional family found in April, but data for April are not included here due to loss of some samples during shipment (see Material and Methods). Hence, the calculation of % richness is based on the total of 214 families.

**Month**	**Detritivo**- **rous**	**Fungivo**- **rous**	**Herbivo**- **rous**	**Omnivo**- **rous**	**Palynivo-rous/ nectarivo-rous**	**Predaceous**	**Sapro**- **xylopha**- **gous**	**Total**
January	8 (41)	3 (171)	18 (1267)	9 (215)	5 (29)	29 (384)	-	72 (2107)
February	3 (25)	3 (157)	18 (819)	9 (297)	11 (38)	29 (370)	1 (7)	74 (1713)
March	12 (570)	4 (355)	21 (708)	12 (496)	21 (246)	36 (581)	1 (3)	107 (2959)
April	Some samples lost during shipment; data not comparable - excluded							
May	12 (1337)	6 (329)	28 (985)	15 (1766)	32 (653)	39 (1163)	1 (1)	133 (6234)
June	12 (1917)	6 (220)	25 (715)	11 (461)	27 (598)	42 (1409)	-	123 (5320)
July	5 (37)	4 (209)	26 (511)	11 (368)	20 (74)	36 (1980)	-	102 (3179)
August	5 (61)	4 (316)	26 (803)	13 (203)	14 (193)	31 (1447)	-	93 (3023)
September	9 (147)	3 (470)	20 (933)	12 (624)	19 (721)	34 (2021)	1 (5)	98 (4921)
October	8 (250)	3 (8)	17 (1545)	8 (131)	17 (308)	25 (1105)	1 (2)	79 (3349)
November	9 (314)	1 (2)	18 (1586)	9 (202)	9 (187)	26 (1176)	1 (8)	73 (3475)
December	8 (93)	2 (232)	17 (708)	8 (200)	8 (157)	33 (561)	1 (1)	77 (1952)
**Total**	**20 (4794)**	**9 (2469)**	**44 (10580)**	**21 (4963)**	51* (3204)	**67 (12197)**	**2 (27)**	214* (38232)
% **richness**	**9.35**	**4.21**	**20.56**	**9.81**	**23.83**	**31.31**	**0.93**	-
% **abundance**	**12.53**	**6.46**	**27.67**	**12.98**	**8.38**	**31.90**	**0.07**	-

**Table 3.** Number of invertebrate families (and individuals) per site and month. - = no specimens were recorded in that group. * one additional family found in April; ** two additional families found in April; ° less effort, data not comparable with other months and sites.

**Month**	**AB**	**CM**	**LS**	**VE**	**SP**	**Total**
January	33 (474)	36 (854)	39 (412)	47 (367)	7 (361)°	72 (2468)
February	37 (196)	34 (666)	40 (469)	40 (382)	3 (4)°	76 (1717)
March	60 (494)	61 (682)	59 (948)	57 (835)	18 (25)	108 (2984)
April	Some samples lost during shipment; data not comparable - excluded					
May	93 (1315)	75 (1014)	76 (2439)	79 (1466)	77 (880)	133 (7114)
June	82 (1400)	73 (975)	80 (1400)	77 (1545)	23 (62)°	123 (5382)
July	64 (581)	54 (722)	59 (1110)	56 (766)	8 (12)°	102 (3191)
August	58 (671)	46 (612)	55 (880)	63 (860)	42 (542)	95 (3565)
September	68 (1066)	61 (917)	63 (1575)	62 (1363)	57 (1217)	99 (6138)
October	43 (407)	36 (1707)	40 (628)	52 (607)	-	79 (3349)
November	50 (535)	39 (1305)	42 (759)	46 (876)	3 (52)°	73 (3527)
December	48 (353)	39 (535)	42 (509)	41 (555)	7 (8)°	77 (1960)
**Total**	159* (7492)	144* (9989)	153* (11129)	146** (9622)	111* (3163)	214* (41395)

**Table 4.** Number of invertebrate families per site and class (all months excluding April). - = no specimens were recorded in that group. * one additional family found in April; ** two additional families found in April; ° less effort, data not comparable with other sites.

**Class**	**AB**	**CM**	**LS**	**VE**	**SP**°	**Total**
Arachnida	34	34	36	42*	27	50
Chilopoda	-	2	1	-	-	3
Diplopoda	1	1	1	-	1	1
Entognatha	4	2*	1*	1	-	4
Gastropoda	10	5	6	5	6	11
Insecta	108*	98	106	96*	75*	143*
Malacostraca	2	2	2	2	2	2
**Total**	159*	144*	153*	146**	111*	214*

**Table 5.** Number of vertebrate species and observations (in brackets) recorded per month. Site SP was excluded because not all methods could be applied at site SP due to restricted accessibility to the swimming pool complex (i.e. no access outside staffed hours, no photo or video recordings possible, no permanent installation of expensive equipment). - = no observations were made in that group.

**Month**	**Birds**	**Mammals**	**Reptiles**	**Amphibians**	**Total**
January	32 (360)	11 (504)	-	-	43 (864)
February	30 (340)	14 (489)	2 (2)	-	46 (831)
March	29 (376)	14 (540)	1 (1)	-	44 (917)
April	27 (198)	14 (505)	1 (1)	-	42 (704)
May	23 (168)	17 (442)	2 (5)	1 (1)	43 (616)
June	22 (194)	18 (499)	4 (13)	-	44 (706)
July	23 (135)	13 (387)	3 (13)	-	39 (535)
August	25 (126)	14 (357)	3 (6)	-	42 (489)
September	19 (130)	16 (313)	3 (5)	-	38 (448)
October	20 (122)	14 (327)	2 (5)	-	36 (454)
November	32 (261)	14 (374)	4 (5)	-	50 (640)
December	28 (344)	13 (345)	1 (1)	-	42 (690)
**Total**	**61 (2754)**	**20 (5082)**	**5 (57)**	**1 (1)**	**87 (7894)**

**Table 6.** Number of vertebrate species (and observations) recorded per site. Site SP was excluded because not all methods could be applied at site SP due to restricted accessibility to the swimming pool complex (i.e. no access outside staffed hours, no photo or video recordings possible, no permanent installation of expensive equipment). - = no observations were made in that group.

**Study site**	**Birds**	**Mammals**	**Reptiles**	**Amphibians**	**Total**
AB	38 (729)	16 (1163)	4 (35)	1 (1)	59 (1928)
CM	33 (649)	13 (1001)	1 (1)	-	47 (1651)
LS	39 (811)	16 (824)	3 (16)	-	58 (1651)
VE	36 (565)	13 (2094)	1 (5)	-	50 (2664)
