# Supplementary material for: Ornamental Phoenix palm trees as habitat for fauna in the Mediterranean Region – results from a full year monitoring
Source: Biodivers Data J. 2024 May 17;12:e123144. doi: 10.3897/BDJ.12.e123144 (PMC11128036; doi:10.3897/BDJ.12.e123144)
Supplement: Supplementary material 1 — Description of Study Sites [file bdj-12-e123144-s001.pdf]

	AB	CM	LS	VE	SP
**Study Site**	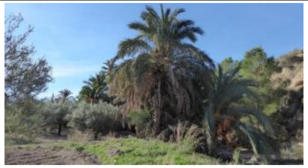	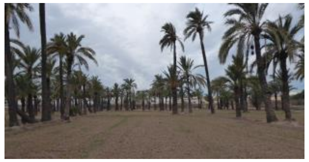	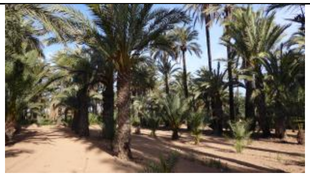	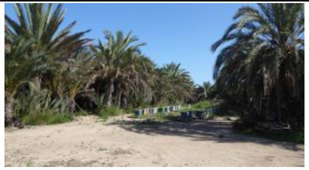	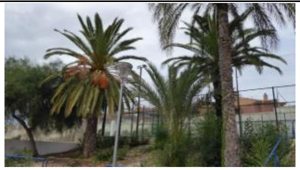
	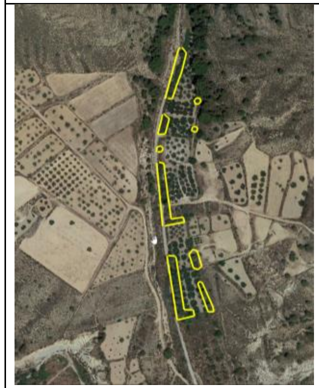	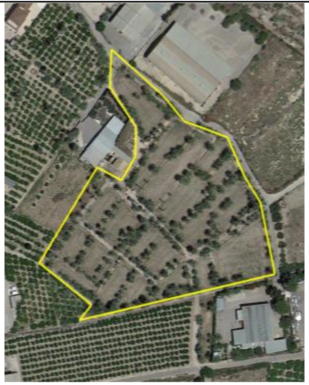	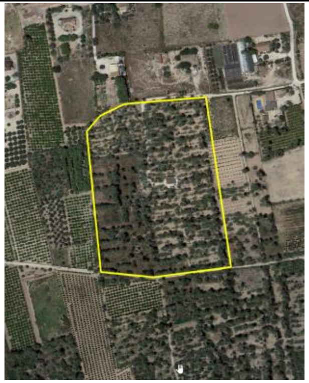	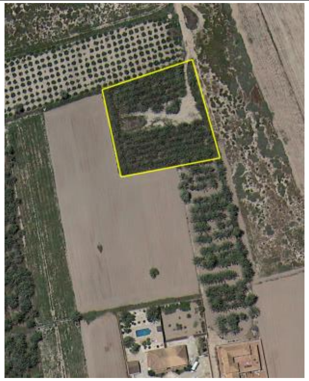	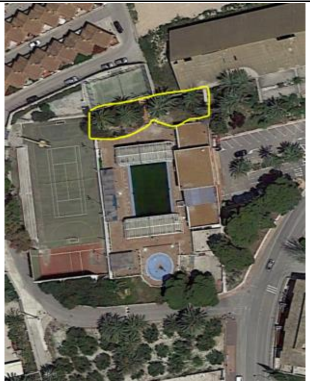
**Coordinates: WGS 84**	38.249465 N<br>1.05016 W	38.239858 N<br>0.714626 W	38.211721 N<br>0.76441 W	38.184913 N<br>0.693135 W	38.201358 N<br>1.043382 W
**Dimensions of study site [m x m] (yellow frame)**	313 x 28	117 x 70	242 x 170	55 x 55	16 x 6
**Number of palm trees in study site**	~100	~400	~1500	314	6
***Phoenix* species**	*P. dactylifera*	*P. dactylifera*	*P. dactylifera* (one *P. canariensis*)	*P. dactylifera*	2 *P. canarisiensis* and 4 *P. dactylifera*
**Range of palm heights [m]**	4 - 15	4 - 25	1 - 35	4 - 7	6 - 8
**Range of palm circumference [cm]**	93 - 205	105 - 210	105 - 225	110 - 220	200 - 220

**Undergrowth**	30% reed, 15% thicket, 15% bare soil, 40% dead palm leaves	5% thicket, 5% bare soil, 90% cereal crop	15% shrubs, 70% bare soil, 15% dead palm leaves	5% reed, 20% thicket, 25% bare soil, 50% dead leaves	90% bare soil, 10% ornamental plants
**Topographical setting**	linear above creek	plain	plain	plain	-
**Soil surface**	mix of sandy sedimentary soil with some small rocks	sedimentary soil, not very hard	sedimentary soil, not very hard	hard sedimentary soil	Very hard soil, covered with gravel
**Temporary water**	yes	yes	yes (partial flood irrigation)	no	no
**Management practice**	Pruning a few palm trees and indirect irrigation due to proximity to other crops (olive trees)	Random pruning on almost all palm trees; flood irrigation twice a year, occasional herbicide use (glyphosate 36%) only in irrigation canals and ploughing once a year for growing oats	Winter pruning, fruit are cut and left on the ground to feed pigs, flood irrigation three times a year, fumigation (imidacloprid 20%) with atomiser and spray guns of most of the palm trees once a year (e.g. December 2020 - January 2021). Regular ploughing, at least every two months.	No management	Winter pruning, constant drip irrigation, fumigation 3-4 times a year, clearing and cleaning of the entire zone done by a professional gardener.
