# Supplementary material for: Ornamental Phoenix palm trees as habitat for fauna in the Mediterranean Region – results from a full year monitoring
Source: Biodivers Data J. 2024 May 17;12:e123144. doi: 10.3897/BDJ.12.e123144 (PMC11128036; doi:10.3897/BDJ.12.e123144)
Supplement: Supplementary material 2 — Example photographs of some of the methods [file bdj-12-e123144-s002.pdf]

Example photographs of some of the methods

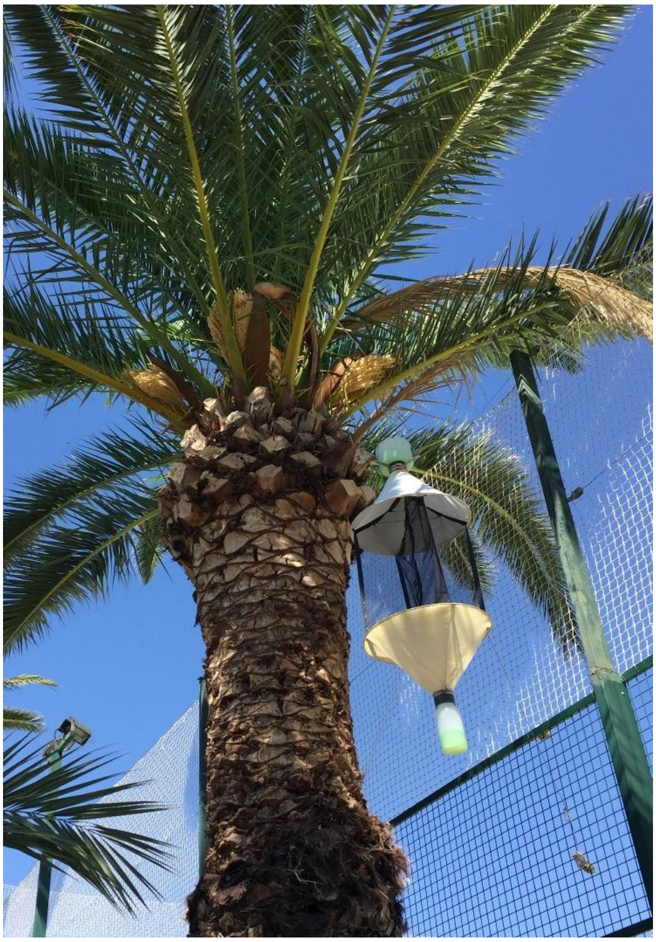

Figure A1: Air eclector ('according to Rahn', Bioform)

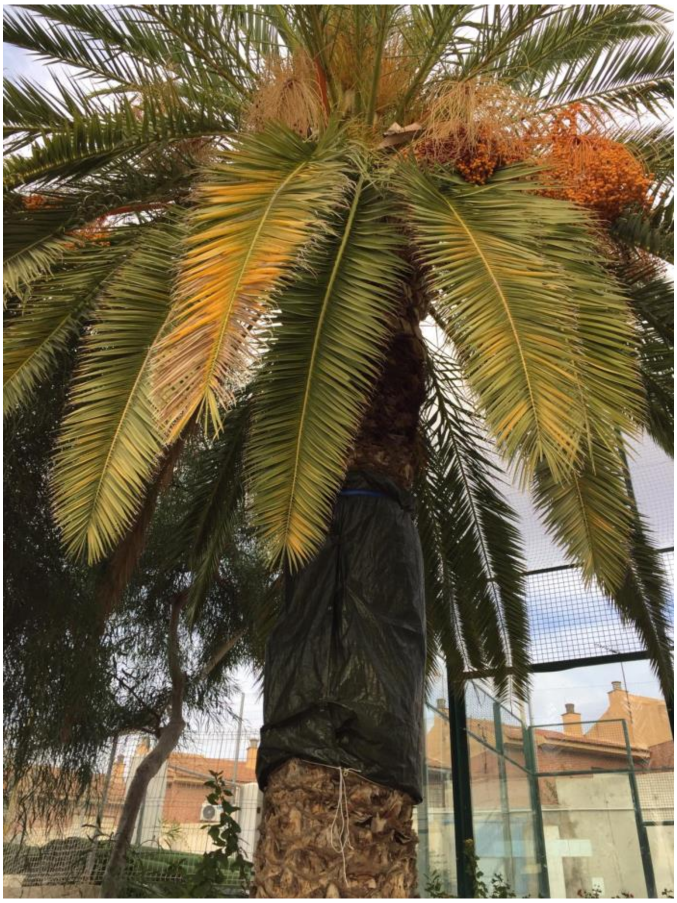

Figure A2: Trunk eclector

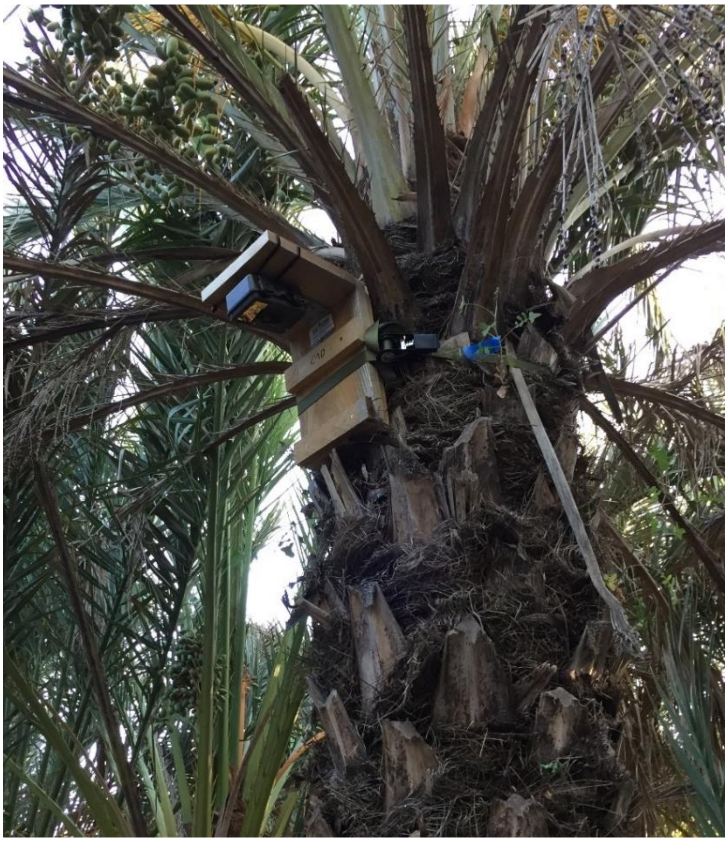

Figure A3: Wildlife camera

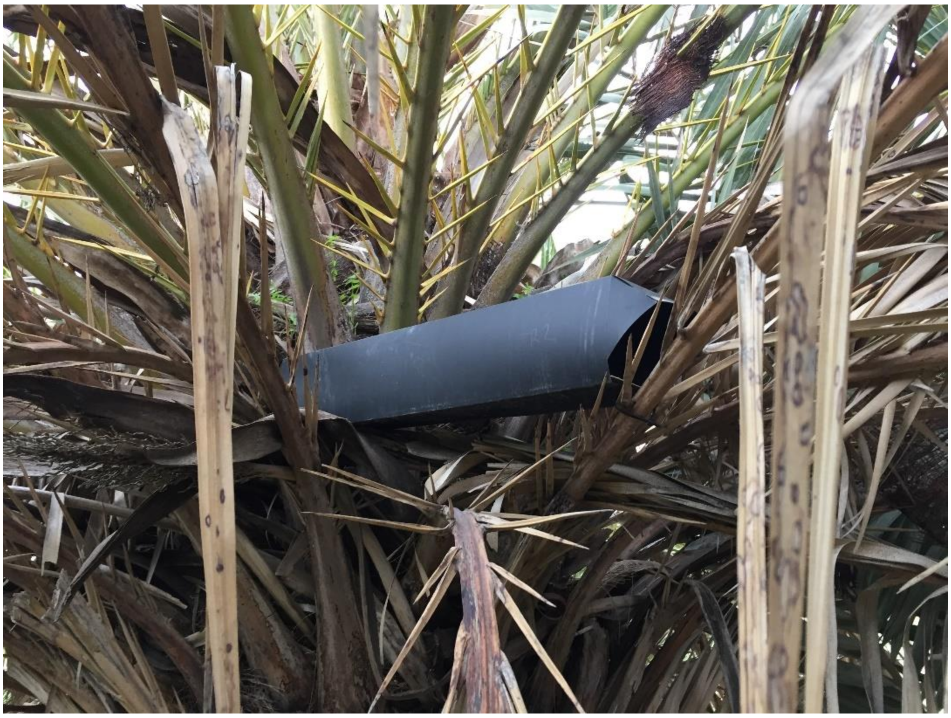

Figure A4: Track tube - crown (Black Trakka monitoring tunnels, Gotcha Traps Ltd.)

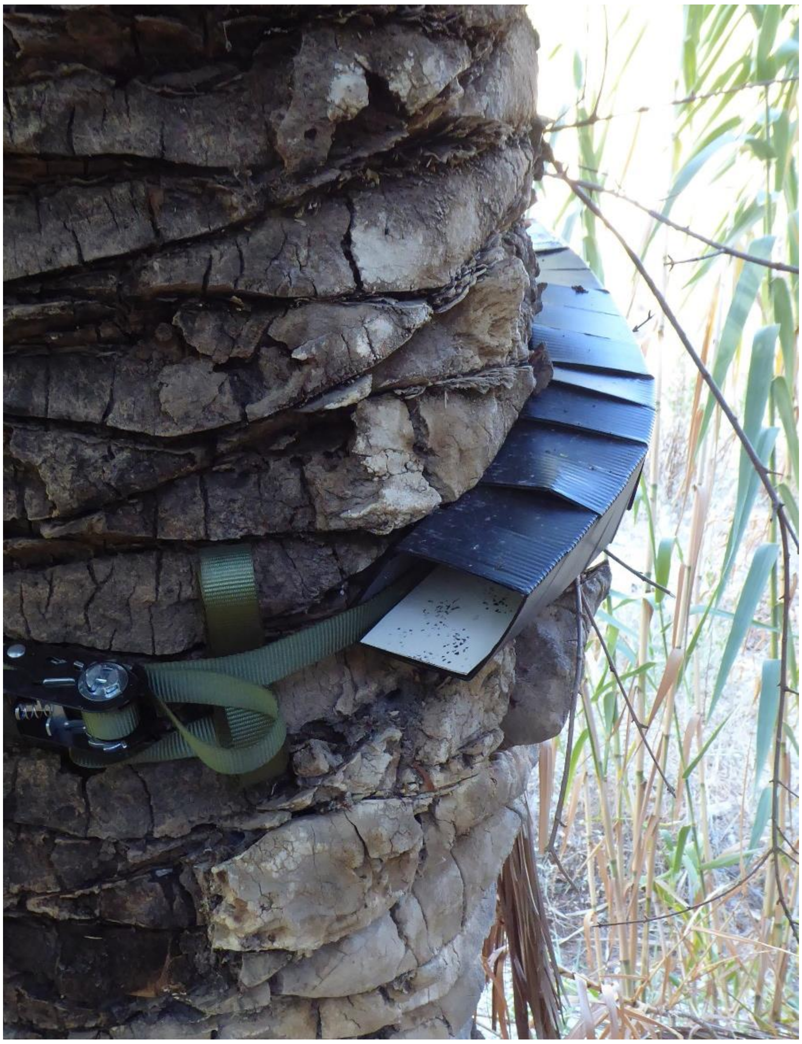

Figure A5: Track tube - trunk

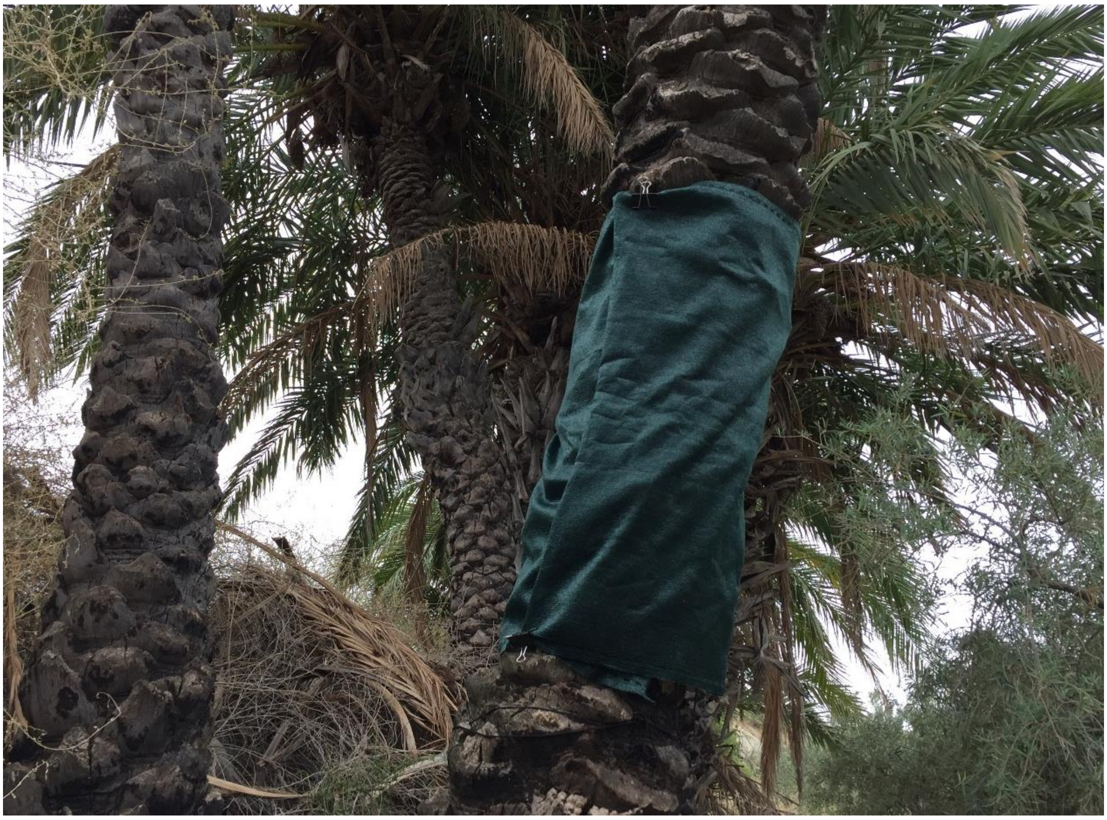

Figure A6: Artificial refuge – arboreal

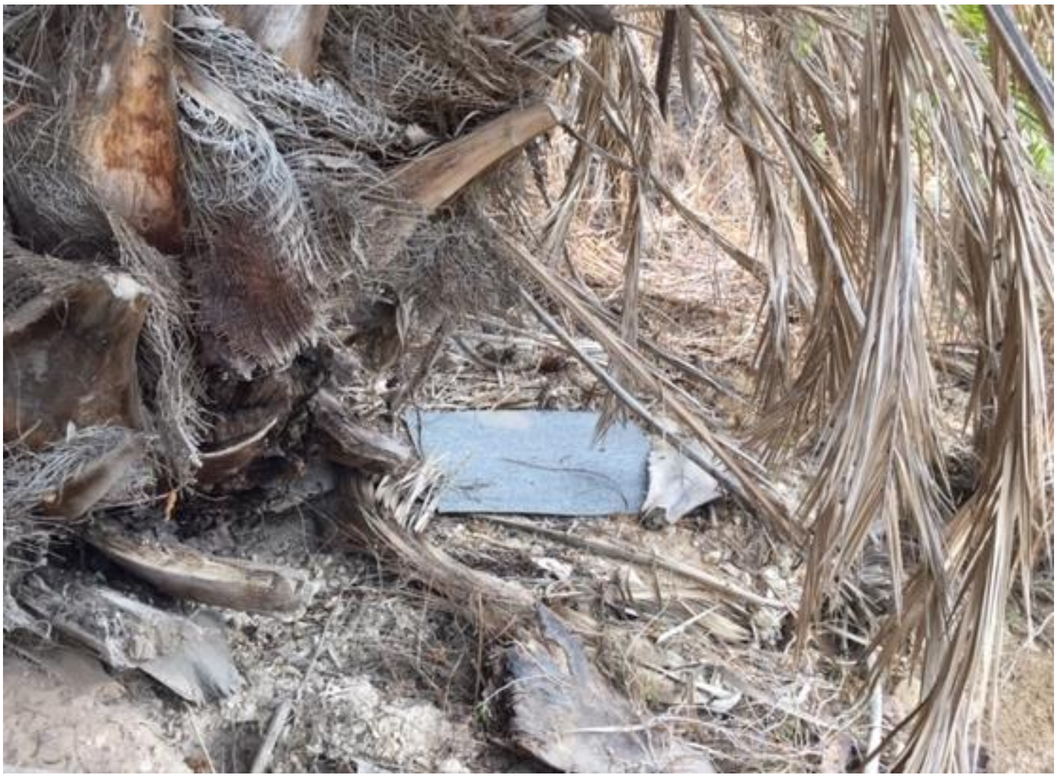

Figure A7: Artificial refuge – terrestrial

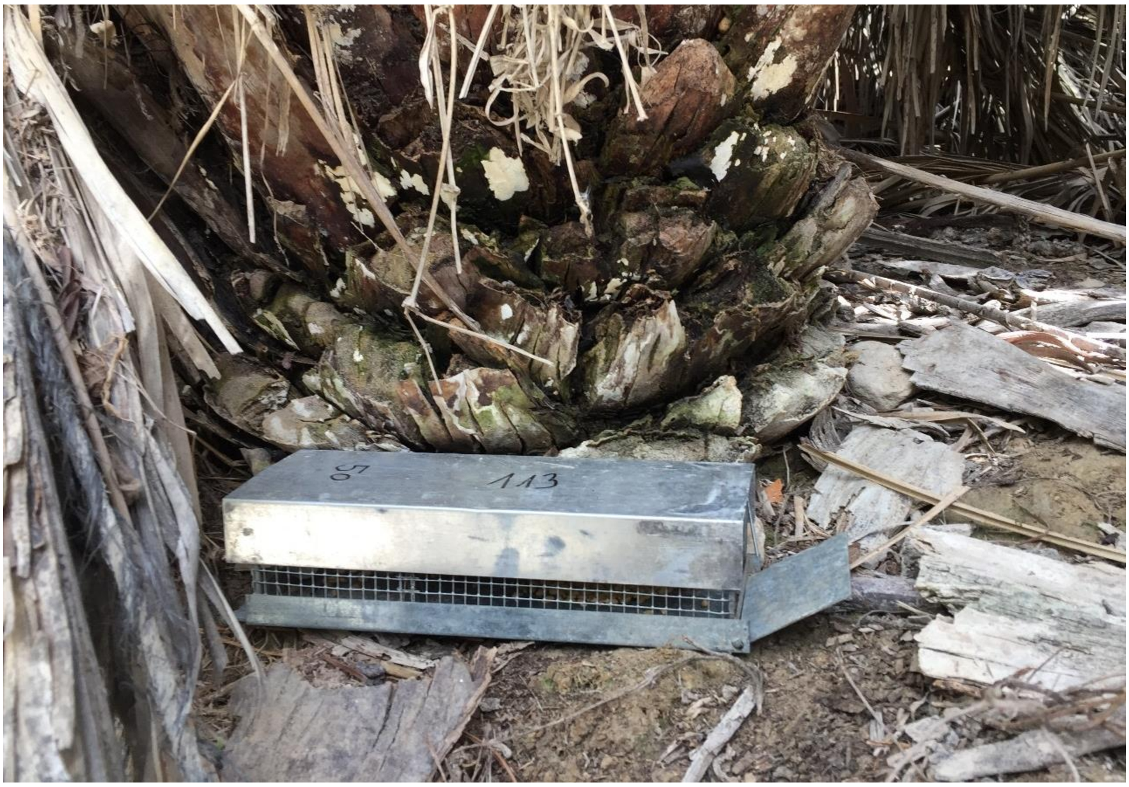

Figure A8: Ugglan small mammal trap (Grahnab)

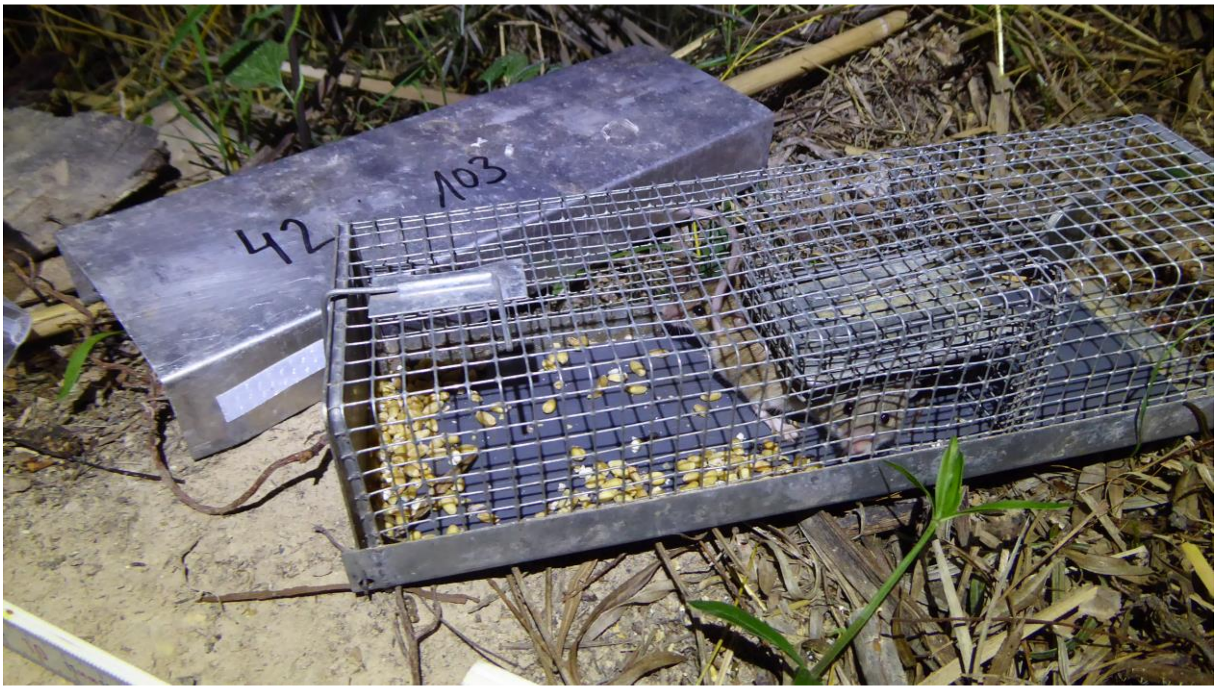

Figure A9: Ugglan small mammal trap (Grahnab) with wood mice (*Apodemus sylvaticus*)
